# Chemotherapy-induced metastasis in breast cancer

**DOI:** 10.18632/oncotarget.22717

**Published:** 2017-11-27

**Authors:** George S. Karagiannis, John S. Condeelis, Maja H. Oktay

**Affiliations:** Maja H. Oktay: Department of Anatomy and Structural Biology, Albert Einstein College of Medicine, Bronx, NY, USA; Gruss-Lipper Biophotonics Center, Albert Einstein College of Medicine, Bronx, NY, USA; Integrated Imaging Program, Albert Einstein College of Medicine, Bronx, NY, USA; Department of Pathology, Montefiore Medical Center, Bronx, NY, USA

**Keywords:** chemotherapy, metastasis, macrophage, dissemination

Randomized prospective studies have indicated that the addition of taxanes into the preoperative, also called neoadjuvant chemotherapy, regimen of breast cancer patients increases the rate of pathological complete response (pCR), but paradoxically does not improve overall survival [1]. Preclinical findings in mouse models of breast and other types of carcinomas suggest that this may be the result of chemotherapy-induced proangiogenic and prometastatic changes in the microenvironment of the primary tumor, triggered as a response to cytotoxic tissue damage [2-4]. It has been well-established that neoadjuvant chemotherapy induces new blood vessel formation as a result of bone marrow progenitor cell infiltration into the primary tumor, including endothelial cell precursors and monocyte/macrophage progenitors, which subsequently provide sufficient support for tumor regrowth [4]. However, the effects of neoadjuvant chemotherapy on tumor metastasis were underexplored until recently.

Indeed, the perivascular proangiogenic macrophages are also capable of assembling specialized microanatomical structures called tumor microenvironment of metastasis (TMEM), known to regulate vascular permeability and cancer cell intravasation and dissemination [5]. In particular, perivascular macrophages expressing high levels of the angiopoietin receptor Tie2 (i.e. Tie2^Hi^ macrophages) secrete high concentrations of vascular endothelial growth factor (VEGF) locally, which in turn disrupts the underlying endothelial junctions and promotes vascular permeability and tumor cell intravasation. Our group has shown that neoadjuvant chemotherapy mobilizes such Tie2^Hi^ macrophages in the primary tumor microenvironment, which significantly promotes TMEM assembly and breast cancer cell dissemination to metastatic sites [3]. Chemotherapy-induced TMEM assembly and metastasis has been further corroborated by another research group using multiple models of breast carcinoma [6].

Neoadjuvant chemotherapy may not only create a metastasis-promoting perivascular microenvironment as described above, but it could also directly affect the phenotypic characteristics and behavior of metastasizing cancer cells. For instance, it has been shown that direct contact of tumor cells and macrophages, an event likely occurring near and at TMEM sites, results in the expression of MENA^INV^, the invasive isoform of the actin-regulatory protein mammalian enabled (MENA) [5]. Indeed, it has been documented that neoadjuvant chemotherapy in preclinical models of breast cancer and in residual tumors from patients after completion of neoadjuvant chemotherapy can significantly increase MENA^INV^-expression [3, 7]. However, the relative amount of Mena^INV^ expression resulting from macrophage-cancer cell contact [3], as opposed to the selection of MENA^INV^ drug-resistant cells [7], remains to be elucidated. In either case, an increase of MENA^INV^ cancer cells in the primary tumor generates a highly invasive and migratory cancer cell subpopulation, capable of TMEM-dependent dissemination and seeding at secondary sites [3, 5].

These preclinical findings strongly suggest that targeting molecular pathways associated with TMEM assembly and TMEM function could serve as an attractive therapeutic strategy to prevent the unwanted side-effect of chemotherapy-induced metastasis. For instance, the selective Tie2 inhibitor rebastinib inhibits TMEM function by inhibiting Tie2 on the TMEM macrophage to prevent VEGF-dependent vascular permeability [8]. The use of rebastinib significantly reduces the number of TMEM-dependent circulating tumor cells in the blood and the number of disseminating tumor cells in the lungs [3], and significantly increases overall survival of paclitaxel-treated mice [8], suggesting that Tie2 inhibition of TMEM alone could confer reversal of the chemotherapy-induced prometastatic tumor microenvironment [3]. Results similar to that obtained with rebastinib inhibition of chemotherapy-induced prometastatic changes have been phenocopied after genetic ablation of the MENA gene in the MMTV-PyMT mouse model, which spontaneously develops metastatic breast tumors [3], consistent with the finding that increased MENA^INV^ expression is a chemotherapy-induced prometastatic change, critical for cancer cell dissemination [3]. Thus, targeting MENA^INV^ may represent another attractive approach for counteracting chemotherapy-induced metastasis.

It appears that chemotherapy-induced proangiogenic and prometastatic changes are not necessarily associated with use of specific drugs or drug families, but represent tissue remodeling associated with a more generalized response to cytotoxic tissue damage [3, 4]. For instance, we have previously demonstrated that TMEM assembly occurs during the course of neoadjuvant treatment irrespective of type of chemotherapy, including taxane (i.e. paclitaxel) and non-taxane (i.e doxorubicin and cyclophosphamide) chemotherapies [3]. Accordingly, stress-inducible genes, such as cyclic AMP-dependent transcription factor ATF3, appear to be non-cancer cell-associated master orchestrators of chemotherapy-exacerbated breast cancer metastasis [6], further confirming a more generalized cytotoxic mechanism behind the chemotherapy-induced pro-metastatic phenotype. However, other researchers have indicated that certain chemotherapeutic drugs (e.g. paclitaxel) can mimic bacterial lipopolysaccharides (LPS), and as such, may signal through tumor cells expressing toll-like receptor-4 (TLR4), thus eliciting their pro-metastatic effects via TLR4-activated systemic pro-inflammatory cytokines [2]. Future research should therefore focus on the precise context discriminating between cancer resistance to specific chemotherapeutics as opposed to chemotherapy-induced non-specific pro-metastatic effects, to help design appropriate treatment modalities.

As noted earlier, the addition of taxanes in preoperative neoadjuvant chemotherapy regimens did not improve metastasis-free or overall survival in breast cancer patients, and in addition, patients who did not achieve pCR may have been at greater risk of developing distant metastasis [1]. Therefore, the studies discussed in this context [2, 3, 6] provide valuable preclinical information, which may help elucidate this clinical paradox. In conclusion, metastasis promoters, such as TMEM and MENA isoforms including MENA^INV^, have the potential to promote the development of pro-metastatic tumor microenvironments in response neoadjuvant chemotherapy and should be investigated further in clinical trials, both as prognostic and predictive markers, as well as therapeutic targets.
